# Comparison of Salt Iodization Requirements in National Standards with Global Guidelines

**DOI:** 10.1093/cdn/nzac116

**Published:** 2022-08-02

**Authors:** Rachel Paige Greenwald, Lana Childs, Helena Pachón, Arnold Timmer, Robin Houston, Karen Codling

**Affiliations:** Emory University, Atlanta, GA, USA; Emory University, Atlanta, GA, USA; Global Fortification Data Exchange, Atlanta, GA, USA; Emory University, Atlanta, GA, USA; Global Fortification Data Exchange, Atlanta, GA, USA; Food Fortification Initiative, Atlanta, GA, USA; Iodine Global Network, Ontario, Canada; Iodine Global Network, Ontario, Canada; Global Fortification Data Exchange, Atlanta, GA, USA; Iodine Global Network, Ontario, Canada

**Keywords:** fortification requirements, WHO guidelines, mandatory fortification, iodine, iodine deficiency, goiter, cretinism

## Abstract

**Background:**

Food fortification is the addition of 1 or more micronutrients to commonly consumed foods and is utilized by many countries as a public health intervention to prevent and control micronutrient deficiencies. As iodine deficiency was a major public health issue globally, the WHO developed evidence-based guidelines for the fortification of salt with iodine. The implementation of salt iodization has been highly successful in reducing iodine deficiency disorders worldwide and is recommended as the main strategy to prevent iodine deficiency.

**Objectives:**

This analysis compared salt iodization requirements specified in countries’ salt standards with WHO 2014 Guidelines on salt fortification.

**Methods:**

For countries with mandatory salt iodization legislation, data from the Global Fortification Data Exchange regarding iodine amounts and iodine compounds, to be added to salt per the country standard and corresponding national salt intake quantities, were compared with 2014 WHO Guidelines.

**Results:**

As of 4 September 2021, 110 countries with mandatory salt iodization legislation had national salt standards that specified iodine amounts and compounds and salt intake data. All but 1 specified at least 1 recommended iodine compound, but the majority specified higher iodine amounts in salt standards than indicated in the guidelines, taking salt consumption levels into account. Our analysis did not find excess iodine intake as a result; however, we did not have data on the extent of compliance with national salt standards.

**Conclusions:**

Existing iodization requirements in salt standards appear to be appropriate for most countries. Countries in which iodine amounts in salt standards are significantly higher than those recommended in the 2014 Guidelines, in particular those with low compliance with national standards or excess iodine intake, may wish to review program process and output indicators and assess whether current iodine amounts in standards would result in excessive intake if implementation was improved.

## Teaser

Although most countries specify higher iodine amounts in salt standards than recommended by WHO, there is no evidence of iodine excess, and most countries follow recommendations for iodine compounds.

## Introduction

Food fortification is a public health strategy that is widely implemented to prevent and control micronutrient deficiencies. Iodine deficiency is one of the most common micronutrient deficiencies and, as of 2015, approximately 2 billion people were estimated to have inadequate iodine intake (1). Iodine is essential for the production of thyroid hormones, which regulate metabolism. As iodine cannot be produced by the body, it must be consumed regularly and in sufficient quantities through the diet (2). However, only a small number of foods are rich in iodine, such as seafood and seaweed; and widespread leaching of iodine from the soil has reduced the iodine content of animal foods, including dairy products. Several populations are therefore unable to obtain sufficient amounts of iodine through their diet (1). Lack of iodine causes increased infant mortality and a range of disorders, such as goiter, cognitive impairment, and neurological disorders, collectively known as iodine deficiency disorders (IDDs) (3). Iodine deficiency in pregnancy can severely impair the fetus's brain and physical development and cause irreversible cognitive deficits in children (2).

In 1994, the WHO and UNICEF recommended the fortification of food-grade salt with iodine (i.e., salt iodization) as the main strategy to achieve IDD elimination (4). Salt is considered a suitable fortification vehicle for several reasons, including because salt is generally consumed by everyone and the technology for salt iodization is simple and cheap to implement (5). Today, 88% of the world's population consume iodized salt (6). Consequently, the number of countries with adequate iodine intake increased from 67 in 1993 to 118 in 2020 (7). In 2014, WHO concluded that iodized salt was effective in reducing the risk of goiter, cretinism, low cognitive function, and iodine deficiency (8).

The WHO, the International Council for the Control of Iodine Deficiency Disorders [ICCIDD; now known as the Iodine Global Network (IGN)], and UNICEF released guidelines for salt iodization in 1994 (4) and 1996 (9), and in 2014, WHO released updated guidelines (5). These guidelines *1*) recommended salt fortification as an efficacious and cost-effective way to achieve optimal iodine nutrition and suggested *2*) iodine amounts to add to salt and *3*) recommended iodine compounds to use for salt fortification. Differences among the guidelines reflect evolving understanding of important factors to consider when setting the amount of iodine in salt. For example, the 1996 Guidelines emphasized considering whether both salt for direct consumption and salt for food processing were to be iodized (9). The 2014 Guidelines emphasized the importance of considering salt intake and suggested different iodine amounts to be added to salt for daily salt intakes from 3–14 g (5). Subsequent iterations of global guidelines have suggested lower iodine amounts in salt. Assuming 10 g salt intake per day, in 1994 the suggested amount of iodine to add to salt was 22–45 mg/kg (4), in 1996 it was 20–40 mg/kg (9), and in 2014 it was 18–22 mg/kg (5). The 2014 Guidelines recommend potassium iodate or potassium iodide as effective iodine compounds for salt fortification; other iodine compounds or forms of iodine are not recommended by WHO (5).

The 2014 Guidelines aim to provide “global, evidence-informed guidelines” to help member states make “informed decisions on the appropriate nutrition actions” (5). In the case of iodine amounts in fortified salt, this evidence includes recommended nutrient intakes (RNIs) for iodine; estimated iodine losses between production and consumption, which vary by salt quality, packaging, and temperature; and iodine bioavailability. Different iodine amounts are recommended based on estimated salt consumption. However, all guidelines are intended to be adapted to particular country contexts. Thus, the 2014 Guidelines emphasize that “concentrations of iodine may need to be adjusted by national authorities” and “the monitoring of urinary iodine concentrations will allow adjustment of the selected fortification concentrations” (5). Approximately 90% of ingested iodine is excreted in urine and therefore median urinary iodine concentration (mUIC) is a reliable indicator of recent iodine intake of a population (5, 10) and, correspondingly, of iodine status (11). As per WHO guidance on the assessment of IDDs, an mUIC in the range of 100–299 μg/L indicates a population has adequate iodine intake (11). In this paper, therefore, reference to iodine intake is based on the indicator mUIC. Iodine amounts in national salt standards, suggested in the 2014 WHO Guidelines, are intended to be adjusted taking into account iodine intake (as measured by urinary iodine excretion or concentration), food-grade salt quality, iodine losses from iodized salt, existence of quality-assurance systems, and household and food industry use of iodized salt (5).

Salt can be categorized into food-grade or edible salt and industrial or nonfood salt. Food-grade/edible salt includes salt for animals and humans. Salt for humans includes table or household salt and salt for food processing. Household/table salt refers to the salt used by households for cooking or at the table. It is usually sold in retail packs, although it can be sold loose (12). Hereafter, we will refer to household/table salt as household salt. Processed food salt is the salt used in the commercial production of processed foods such as bread, cheese, processed meats and fish, convenience foods such as instant noodles, snacks, and condiments such as bouillon cubes, soy sauce, and fish sauce. It is often more refined, of higher quality, and sold in bulk (12). Recognizing that WHO Guidelines reflect the best available advice on iodine amounts and compounds for salt fortification and could be expected to be the starting point for development of national salt fortification requirements, the goal of this study was to compare salt iodization requirements with WHO Guidelines.

## Methods

This study was a comparison of salt iodization requirements—iodine amounts and compounds—indicated in current country salt standards with WHO 2014 Guidelines on salt fortification (5), and was based on the first author's undergraduate honors thesis at Emory University (13). The Emory University Institutional Review Board indicated that no review was necessary because no human subjects were used.

### Data sources

Data for this analysis were downloaded from the Global Fortification Data Exchange (GFDx), a food fortification analysis and visualization tool that acquires data from annual surveys, literature reviews, and regional and national contacts (14). The GFDx has country-level data on fortification aspects of salt and other foods such as legislation, fortification standards, food intake, and proportion of people consuming fortified foods. The GFDx obtained salt intake data (15) for 186 countries from 1990 and 2010 from a systematic analysis of 24-h urinary sodium and dietary sodium country surveys or modeled intake (16) and for seven countries from a review of salt reduction initiatives, which provided data for 2011 and 2012 (17). Iodine fortification requirements in national salt standards are usually expressed as allowed or expected ranges of iodine or a minimum. The GFDx recorded the midpoint of the range, or the single amount indicated in the standard; the unit was standardized to parts per million (ppm) such as milligrams per kilogram (18). The mUIC values, as an indicator of the adequacy of iodine intake, were obtained from the IGN (19).

### Salt iodization requirements in standards

The analysis included countries with mandatory salt fortification legislation and salt standards indicating iodine amounts and allowed iodine compounds. Ideally, this analysis would have been done on salt standards issued or in place at the time that salt iodization started in the country, recognizing that applicable WHO guidelines might be followed more closely at the start of a program. However, since the GFDx compiles current salt standards and does not consistently have initial or previous standards for all countries, this analysis was undertaken using current standards, which are not necessarily the same as the standards issued when salt iodization was first legislated. As the 2014 Guidelines only suggest iodine amounts that should be present at the production or import level, we did not review amounts indicated in national salt standards for retail or household level or other nutrients such as fluoride or iron.

### 2014 Guidelines

The 2014 Guidelines suggest iodine amounts that should be present at the point of production or import, for salt intake between 3 and 14 g/d (**Table 1**) (5) and state that potassium iodate (KIO_3_) or potassium iodide (KI) can be used in salt fortification (5). Regardless of when the national standards were issued, we used countries’ most recent salt intake data in the GFDx (15).

**TABLE 1:** Suggested iodine amounts in WHO 2014 Salt Fortification Guidelines^1^

Suggested concentrations for the fortification of food-grade salt with iodine for different amounts of salt consumption.	
Estimated salt consumption,^2^ g/d	Average amount of iodine to add, mg/kg salt (RNI + losses^3^)
3*	65
4*	49
5*	39
6	33
7	28
8	24
9	22
10	20
11	18
12	16
13	15
14	14

1 The table is reproduced from the 2014 WHO Salt Fortification Guidelines (5). *Corresponds to the WHO salt reduction guideline. RNI, recommended nutrient intake (the daily intake, set at the Estimated Average Requirement plus 2 SDs, which meets the nutrient requirements of almost all apparently healthy individuals in an age- and sex-specific population group).

2 This includes consumption as table salt as well as salt from processed foods.

3 This fortification concentration was calculated based on the mean RNI of 150 μg iodine/d + 30% losses from production to household level before consumption, and a 92% iodine bioavailability. Losses depend on the iodization process, the quality of salt and packaging materials and the climatic conditions. Losses could vary widely (8) and this table presents the value considering 30% losses. The monitoring of urinary iodine concentrations will allow adjustment of the selected fortification concentrations. Although iodate is more stable, either potassium iodate (KIO_3_) or iodide (KI) can be used. Iodide may be used for dry, low crystal size and washed or refined salt. While iodate can be used alone and in any type of salt quality, iodide is used in very good quality salt and cannot be added alone. Therefore, some salt producers add sodium carbonate or sodium bicarbonate when they iodize salt, to increase alkalinity, and sodium thiosulfate or dextrose to stabilize potassium iodide. Without a stabilizer, potassium iodide may be oxidized to iodine and lost by volatilization from the product [The Salt Institute. Iodized salt. Available from: http://www.saltinstitute.org/news-articles/iodized-salt/ (accessed 2014 Sep 10)]. An estimated additional variability of ±10% during iodization procedures could be considered at the production site for use in quality-control and -assurance procedures. This variability depends on the iodization methods used and quality assurance system in place.

For iodine amounts, the 2014 Guidelines provide recommendations for the “average amount of iodine to add” (5). As salt standards specified iodine fortification requirements as allowed or expected ranges or a minimum, the GFDx recorded the midpoint of the range, or the single amount indicated in the standard (18). In this analysis, we compared the recommended iodine amount in the 2014 Guidelines with the midpoint or single amount recorded by the GFDx. The 2014 Guidelines also assume all food-grade salt—household/table salt as well as salt from processed foods—is iodized (5).

### Comparison of iodine amounts required in national salt standards with those in 2014 Guidelines (calculated as percentage of 2014 Guidelines met)

We undertook the analysis for countries with mandatory legislation and standards that indicated both iodine amounts and iodine compounds and had salt intake data. We extracted the midpoint of iodine ranges or single amount in national standards, as recorded in the GFDx (18), and the suggested iodine amount in the 2014 Guidelines into Excel (Microsoft Corporation). We then divided the amount of iodine recorded by the GFDx by the suggested amount in the 2014 Guidelines, and multiplied by 100 to obtain the percentage of 2014 Guidelines met. One hundred percent means the iodine requirement from the national standard recorded by the GFDx was the same as the amount recommended by the 2014 Guidelines; 50% means that the iodine requirement was half the amount recommended by WHO, 200% means the requirement was double the WHO-recommended amount, and so forth. For example, country X requires 30–50 ppm of iodine in its salt standard; the midpoint is 40 mg/kg. The estimated salt intake in country X is 10 g/d. Based on salt intake, the 2014 Guidelines suggest the addition of 20 mg iodine to salt. The comparison between the iodine requirement indicated in salt standards and 2014 Guidelines was then calculated as follows: 40/20 = 2.0 × 100 = 200%. This country's national requirement was classified as 200% more than the WHO-recommended amount (double the WHO-recommended amount).

We then extracted from the GFDx the scope of national salt iodization legislation (20) and categorized countries as requiring both household AND processed food salt to be iodized or those requiring only household OR processed food salt to be iodized and compared the scope with the percentage of 2014 Guidelines met. The 2014 Guidelines assume all food-grade salt is iodized—that is, both household salt and processed food salt; however, if a country only requires iodization of household salt or processed food salt, it may have elected to require higher amounts of iodine in national salt standards.

### Comparison of allowed iodine compounds in national salt standards with those in 2014 Guidelines

We extracted the allowed iodine compounds in national standards, as recorded in the GFDx (18), and the recommended compounds from the 2014 Guidelines (potassium iodate or potassium iodide) into Excel. Countries were then classified as follows: *1*) all of the compounds in the country's standard are WHO-recommended compounds, *2*) there are both WHO-recommended compounds and nonrecommended compounds, or *3*) all of the compounds in the country's standard are non–WHO-recommended compounds.

### Comparison of percentage of 2014 Guidelines met with iodine intake

We then assessed if the percentage of 2014 Guidelines met (i.e., the difference between iodine amounts required in national salt standards and those in 2014 Guidelines) was associated with iodine intake (as measured by mUIC). Ideally, we would have compared the percentage of 2014 Guidelines met with iodine intake in countries where the iodine amount in iodized salt complies with salt standards; however, such compliance data are not readily available (21). A widely available alternative is the proportion of households with salt iodized to any level (6); this is evidence that salt is being iodized but not if it complies with salt standards at the time of production. Thus, we compared mUIC and the percentage of 2014 Guidelines met only in countries where ≥70% of households were consuming salt iodized at any level from a household survey that was undertaken 1–5 y before mUIC was assessed and after (>1 y) the salt standard was issued. Countries meeting these criteria were fortifying the majority of salt (>70% of households) presumably as per the national standard (>1 y after the standard was issued), and in the period (1–5 y) before mUIC was assessed, such that compliance with the standard could be expected to determine iodine intake status. For example, country Y's mUIC was assessed in 2017. Country Y had 81.5% of households consuming salt iodized at any level from a household survey that was undertaken in 2012–2013, which occurred 1–5 y before mUIC was assessed and after their salt standard was issued in 2000. Country X met our criteria and the percentage of the 2014 Guidelines met was then compared with the country's iodine intake. As these criteria yielded a relatively small sample size (*n* = 15), we repeated the analysis for all countries with a calculated percentage that met of the 2014 Guidelines and which had mUIC in school-age children, measured after mandatory salt fortification became effective (*n* = 74). mUIC in schoolchildren was used as the largest number of countries have assessed mUIC in this population group because of their combined high vulnerability, easy access, and applicability to a variety of surveillance activities (11).

In this case, we made the assumption that salt iodization requirements in salt standards were being followed. The Pearson correlation coefficient was calculated using SAS (SAS Institute) to see if any correlations were statistically significant.

## Results

### Countries in the analysis

**FIGURE 1:**
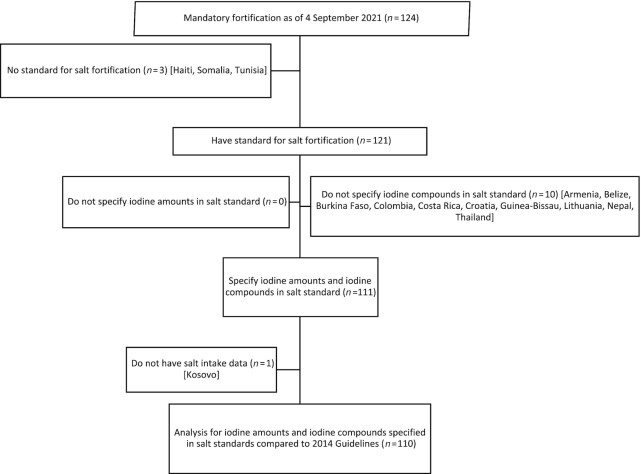
Flowchart of countries with mandatory salt fortification included in the comparison of iodine amounts and iodine compounds in standards with 2014 Guidelines for salt fortification (22).

The 2014 Guidelines recommend salt fortification to achieve optimal iodine intake (5). Out of 196 countries (14) and as of 4 September 2021, 124 (63%) have mandatory legislation for salt iodization (22). All of the countries with mandatory legislation were included in the analysis if the GFDx had a salt standard for them, and if it specifies iodine amounts and iodine compounds. Countries were also excluded from the analysis if the GFDx lacked salt intake data (**Figure 1**).

### Comparison of salt iodization requirements for iodine amounts with 2014 Guidelines

**FIGURE 2:**
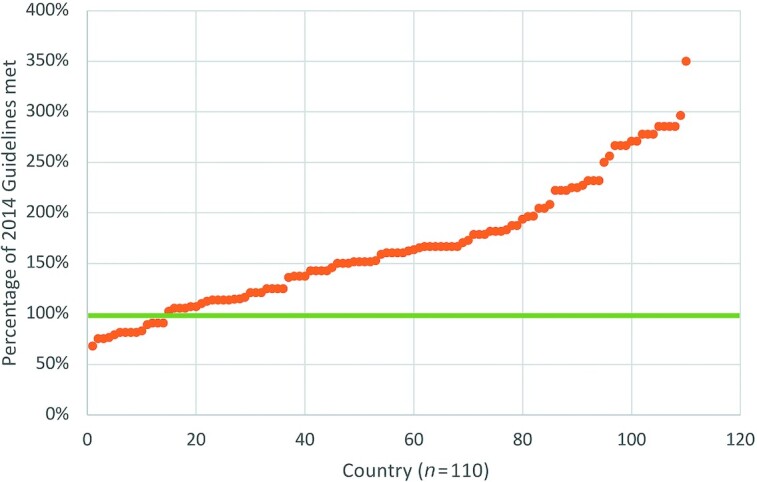
Comparison of iodine amounts required in national salt standards with those in 2014 Guidelines, taking into consideration salt intake levels (*n*  = 110). Note: The orange dots represent the iodine amounts in salt standards as a percentge of the 2014 WHO requirements. The green line (100%) indicates that the amount of iodine req uired in the country's salt standard was equal to the amount of iodine recommended by the 2014 Guidelines. If a country was classified as below 100%, the country's standard was less than 2014 Guidelines. If a country was classified as above 100%, the country's standard was more than 2014 Guidelines.

**FIGURE 3:**
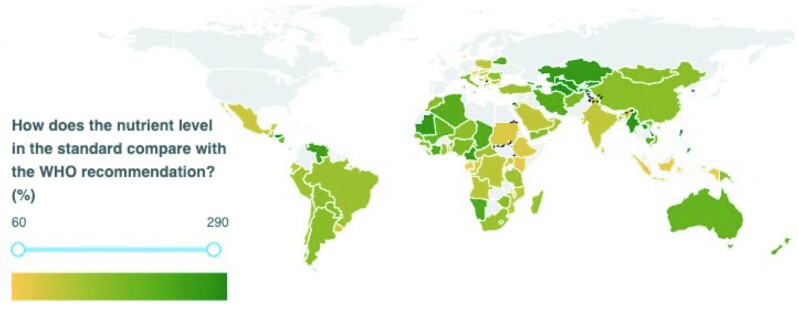
Comparison of iodine amounts required in national salt standards when using potassium iodate with 2014 Guidelines, taking into consideration salt intake levels (*n* = 104). Note: Countries in gray were excluded if they did not have mandatory legislation, did not have a standard, or did not have a standard that specified iodine amounts, or lacked salt intake data. GFDx, Global Fortification Data Exchange. [Map obtained from the GFDx (23)].

**FIGURE 4:**
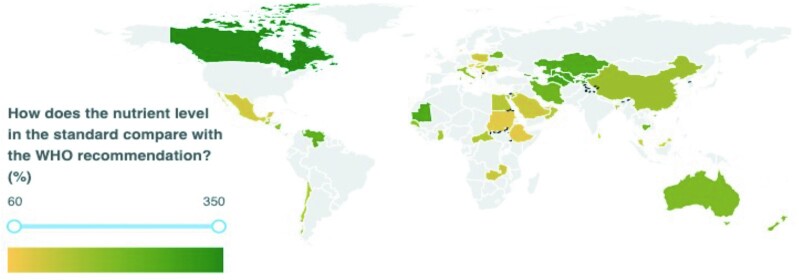
Comparison of iodine amounts required in national salt standards when using potassium iodide with 2014 Guidelines, taking into consideration salt intake levels (*n* = 55). Note: Countries in gray were excluded if they did not have mandatory legislation, did not have a standard, or did not have a standard that specified iodine amounts, or lacked salt intake data. GFDx, Global Fortification Data Exchange. [Map obtained from the GFDx (23)].

Of the 110 countries included in this analysis, iodine amounts in salt standards fell below the level recommended by the 2014 Guidelines in 13% of countries and were higher in all remaining countries, as shown in **Figure 2**. The percentage of 2014 Guidelines met ranged from 68% (Serbia) to 350% (Canada). The results of our analysis by country are shown in **Figure 3** for potassium iodate and **Figure 4** for potassium iodide. Both maps were obtained from the GFDx (23).

**FIGURE 5:**
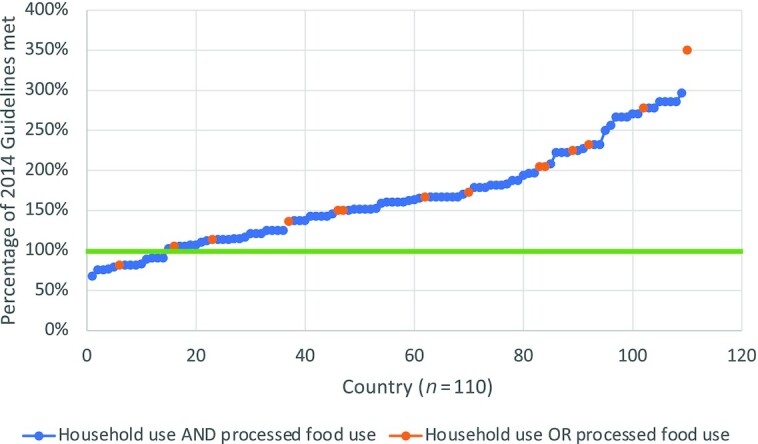
Comparison of iodine amounts in national salt standards with WHO 2014 Guidelines, taking into consideration scope of the salt iodization legislation (*n* = 110). Note: The green line (100%) indicates that the amount of iodine required in the country's salt standard was equal to the amount of iodine recommended by the 2014 Guidelines. If a country was classified as below 100%, the country's standard was less than 2014 Guidelines. If a country was classified as above 100%, the country's standard was more than 2014 Guidelines.

We then conducted the same analysis taking legislation scope into account. In 96 countries both household salt AND salt for processed food are required to be iodized, whereas in 14 countries only household use OR processed food use salt must be iodized. We found no association between legislation scope and the percentage of 2014 Guidelines met. While it might be expected that iodine amounts in national salt standards might be higher when only salt for household use or salt for processed food use is required to be iodized, we found that countries requiring the iodization of either household or processed food salt were spread relatively evenly across the range of percentage of 2014 Guidelines met, as shown in **Figure 5**.

### Comparison of allowed iodine compounds in national salt standards with 2014 Guidelines–recommended compounds

**FIGURE 6:**
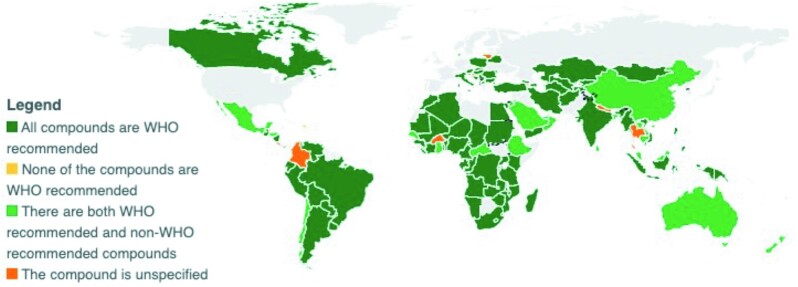
Comparison of allowed iodine compounds in national salt standards with WHO 2014 Guidelines (*n* = 110). Note: Countries in gray are excluded because they do not have mandatory salt fortification legislation, or they have no standards available. GFDx, Global Fortification Data Exchange. [Map obtained from the GFDx (24)].

Of the 110 countries included in this analysis, 74% of countries’ standards included only 2014 Guidelines–recommended compounds, 25% included both 2014 Guidelines–recommended and nonrecommended compounds, and 1% included only non–2014 Guidelines–recommended compounds, as shown in **Figure 6**, a map obtained from the GFDx (24).

### Is there evidence of high mUIC in countries where iodine amounts in country standards are higher than 2014 WHO Guidelines?

**FIGURE 7:**
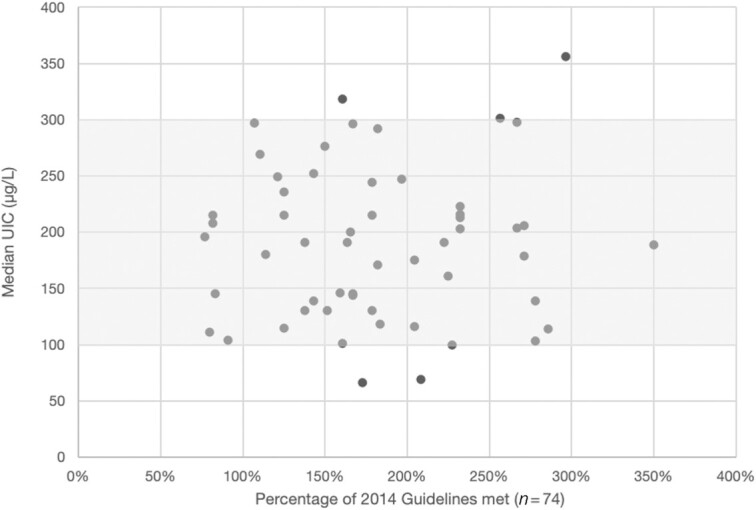
Comparison of the percentage of 2014 Guidelines met with iodine intake data after mandatory fortification became effective (*n* = 74). Note: Comparison was undertaken for all countries for which we had calculated the percentage of the 2014 Guidelines met and which had mUIC in school-age children assessed after salt iodization was made mandatory. The shaded box indicates adequate iodine intake as reflected by mUIC, in the range of 100–299 µg/L. mUIC, median urinary iodine concentration; UIC, urinary iodine concentration.

Only 15 (14%) countries included in the analysis met our criteria of *1*) having national data on mUIC in the general population (school-age children and women of reproductive age) after the standard was issued and *2*) household use of iodized salt assessed 1–5 y prior to the mUIC data and *3*) showing >70% use of iodized salt. mUIC in the range 100–299 μg/L defines this population as having adequate iodine intake. Within this small sample, all of the countries had iodine amounts in salt standards calculated to be higher than 2014 Guidelines—ranging from 110% to 286% higher. However, of these 15 countries, all but 3 had adequate iodine intake. One country had excessive iodine intake; it had iodine amounts in its national standard that were calculated to be 256% higher than the 2014 Guidelines. Two countries had inadequate iodine intake; they had iodine amounts in their national standards that were calculated to be 222% and 286% higher than the 2014 Guidelines, respectively. As such, having iodine amounts in salt standards in excess of 2014 Guidelines did not appear to increase the likelihood of excessive iodine intake. Similarly, we found no correlation (*r* = –0.04923; *P* = 0.6770) when we increased the sample size to 74 countries by simplifying our criteria to include all countries with mUIC data in school-age children measured after legislation for mandatory salt fortification was passed (**Figure 7**).

## Discussion

Most countries follow the 2014 WHO Guidelines in having mandatory legislation for fortification of salt with iodine. The majority of countries with mandatory fortification follow 2014 Guidelines regarding iodine compounds; 74% allow only potassium iodate and/or potassium iodide, 1 country did not allow any WHO-recommended compounds, and the remainder allow both WHO-recommended and nonrecommended compounds. In contrast, the iodine amount in national standards was calculated to be higher than 2014 Guidelines in 87% of countries. However, after comparing iodine intake, as measured by mUIC, with the percentage met of 2014 Guidelines, we found no indication that having iodine amounts in salt standards above those indicated in the 2014 Guidelines was correlated with excessive iodine intakes, even when iodine amounts in salt standards were calculated to be 2 or more times greater than suggested. Similarly, higher iodine amounts in national salt standards did not seem to be explained by legislation scope. We expected to see countries who require iodization of only table OR food industry salt to have higher amounts of iodine in national standards compared with the countries who require iodization of both table AND food industry salt; however, we saw no association between legislation scope and countries with higher requirements for iodine than the 2014 Guidelines.

### Why iodine amounts in salt standards may not align with 2014 Guidelines

The 2014 Guidelines on salt fortification supersede previous guidelines and take into consideration all available global evidence and lessons learned on salt iodization to guide countries in achieving optimal iodine intake through salt fortification with iodine (5). It is intended that countries should look to these 2014 Guidelines as a starting point when setting iodization requirements and make necessary adaptations based on the country context. As national salt iodization programs are implemented, it is intended that key implementation indicators are monitored, including the proportion of households consuming adequately iodized salt and/or the extent to which iodized salt is used in the production of processed foods. When such monitoring indicates that the program is being adequately implemented, it is appropriate to assess program impact on iodine intake. If iodine intake, as usually measured by mUIC, is found to be excessive or insufficient despite optimal implementation of the program, it would be appropriate to amend requirements for salt iodization in national salt standards to adjust the amount of additional iodine being delivered by the program. Thus, iodine amounts in current national standards may vary from amounts indicated in the 2014 Guidelines because of adaptations made by the country when first developing national iodization requirements and/or adaptations made subsequently in response to program impact indicators—in particular, iodine intake.

National salt iodization requirements may not follow 2014 Guidelines if they follow other guidance. For example, the following regional groupings have regional resolutions or standards that include specification of iodine amounts to add to salt and allowed iodine compounds: Commonwealth of Independent States (25), Gulf Cooperation Council (26), Eurasian Economic Union (27), Economic Community of West African States (28), and East African Community (29). Meanwhile, the European Union indicates allowed iodine compounds but not iodine amounts (30).

Prior to the 2014 Guidelines on salt fortification, recommendations were issued in 1994 (4) and 1996 (9) and these earlier guidelines recommended higher amounts of iodine. Thus, countries with standards issued in the 1990s and 2000s might have followed previous WHO guidelines and have higher iodine addition amounts than recommended by the 2014 Guidelines.

### Interpretation of the findings

While countries broadly followed 2014 Guidelines for salt fortification, a majority required higher iodine amounts than theoretically needed. However, this analysis did not find evidence of excess iodine intake as a result. Higher than recommended iodine amounts in standards may not be causing excess iodine intakes for a number of reasons including the following: *1*) countries have set iodine amounts based on national context including alternative data on salt intake, *2*) there is poor compliance with and/or enforcement of national standards and actual iodine amounts in salt are lower than specified in standards, and *3*) if population coverage of fortified salt is not universal, national iodine intake may mask pockets of inadequate and/or excess iodine intake. However, if implementation was improved in these countries such that salt was universally iodized and compliant with national standards, iodine amounts in current standards might lead to excessive intake. Therefore, countries in which salt iodization requirements are significantly higher than those recommended in the 2014 Guidelines—in particular, those with low compliance with national standards (i.e., some salt is not fortified or iodine content is less than required by national standards) or excess iodine intake—may wish to review program process and output indicators and assess whether current iodization requirements are optimal for the country context.

### Comparison with other analyses

A similar analysis utilized the same methodology to compare countries’ fortification standards for wheat and maize flour with WHO flour fortification guidelines for up to 10 nutrients. The analysis generally found good alignment for fortification compounds and lower-than-recommended amounts of iron, zinc, and vitamin B-12 (31). This and our analysis indicate that countries might not be following WHO guidelines for other nutrients besides iodine and for other foods besides salt. To our knowledge, these studies on wheat flour and salt are the first comparisons of food-fortification requirements with WHO fortification guidelines.

### Strengths and weaknesses

A strength of this study is that the data came from the GFDx, which has comprehensive and vetted data on salt fortification for 196 countries (14) and includes information from all countries regardless of the language of fortification documents (32). Another strength is that 89% of countries with mandatory legislation for salt fortification were included in the analysis. Finally, the analysis included data from 3 different sources: the GFDx (14), UNICEF (6), and IGN (19).

This study has several limitations. We were not able to take into account information that may have been used to develop national iodization requirements. The analysis compares national iodization requirements in salt standards with iodine amounts suggested in 2014 Guidelines. However, 2014 Guidelines themselves recommend adjustments based on national context, such as actual iodine losses from iodized salt or iodine intake. Similarly, our analysis was unable to take into consideration the implementation or enforcement of the national salt standards in these countries because there are few available compliance data (21). Without these data, we do not know whether iodine amounts in salt standards are reflective of actual iodine amounts at a production or import level. We were unable to adequately assess whether iodine amounts in salt standards in excess of iodine amounts suggested by the 2014 Guidelines were contributing to excess iodine intake because of a lack of data on household and processed foods’ use of salt iodized in compliance with national salt standards. We were only able to do this analysis for household use of salt with “any iodine,” which does not sufficiently reflect compliance with national standards. Additionally, we have predominantly relied on Powles and colleagues’ (16) data on salt intake when comparing iodine amounts in salt standards with the 2014 Guidelines. While Powles’ study is a systematic analysis, salt intakes were modeled for some countries with missing or limited data. If estimates of salt intake in the GFDx (15), which were used in this analysis, are not reflective of actual national salt intake, our assessment of the alignment of national standards with 2014 Guidelines may be incorrect. Similarly, countries might have used alternative estimates of salt intake when setting their iodine addition amounts. Finally, while the GFDx makes every effort to keep its data up to date and to correct any misinformation, it is possible that the GFDx may not have the most recent salt standard for every country.

### Conclusions

The majority of countries have adopted mandatory salt fortification with iodine and allow for at least 1 recommended iodine compound as per 2014 Guidelines. Eighty-seven percent of countries with mandatory salt fortification require higher amounts of iodine in national standards than are theoretically needed and suggested by 2014 Guidelines. However, comparison with iodine intake data did not find any correlation with excess iodine intake. As such, existing iodization requirements in salt standards appear to be appropriate but countries in which iodine amounts in salt standards are significantly higher than those recommended in the 2014 Guidelines—in particular, those with low compliance with national standards or excess iodine intake—may wish to review program process and output indicators and assess whether current iodine amounts in standards are optimal for the country context. The country that has not specified the use of either of the WHO-recommended compounds may also wish to review their specifications.

## Data Availability

Data described in the manuscript on alignment of national salt fortification requirements with WHO recommendations are publicly and freely available at https://fortificationdata.org/alignment-of-national-fortification-standards-with-who-recommendations-nutrient-compounds/ and https://fortificationdata.org/gfdx-analysis-alignment-of-national-fortification-standards-with-who-recommendations-nutrient-levels/ and comparisons of alignment with iodine status are available upon request from the corresponding author. Conflict of Interest Statement: Co-authors are affiliated with Emory University, the Iodine Global Network, the Global Fortification Data Exchange, and the Food Fortification Initiative. All these organizations help country leaders promote, plan, implement, monitor or evaluate food fortification. Funding Statement: This work was supported, in whole or in part, by the Bill & Melinda Gates Foundation [OPP1210398]. Under the grant conditions of the Foundation, a Creative Commons Attribution 4.0 Generic License has already been assigned to the Author Accepted Manuscript version that might arise from this submission. All authors had full control of primary data.
